# Antibiotic susceptibility of *Pseudomonas aeruginosa* in Saudi Arabia: a national antimicrobial resistance surveillance study

**DOI:** 10.3389/fpubh.2024.1436648

**Published:** 2024-09-30

**Authors:** Abrar K. Thabit, Ammar M. Alghamdi, Musaab Y. Miaji, Feras S. Alharbi, Anas F. Jawah, Fatimah Alturki, Nehal Hosin, Mohammed Bazuqamah, Masaad Saeed Almutairi, Hamad Alhamed, Alaa Elhendawy, Dalya Atallah, Abdulaziz A. Humadi, Khalid A. Alfifi, Khadija Alfadel, Khalid Eljaaly, Mahmoud A. Elfaky, the Saudi AntiMicrobial Surveillance (SAMS) study group

**Affiliations:** ^1^Department of Pharmacy Practice, Faculty of Pharmacy, King Abdulaziz University, Jeddah, Saudi Arabia; ^2^Faculty of Pharmacy, King Abdulaziz University, Jeddah, Saudi Arabia; ^3^Department of Microbiology, King Fahad Hospital of the University, Dammam, Saudi Arabia; ^4^Department of Microbiology, College of Medicine, Imam Abdulrahman Bin Faisal University, Al Khobar, Saudi Arabia; ^5^Department of Microbiology, King Khaled Hospital, Najran, Saudi Arabia; ^6^Department of Pharmacy Practice, College of Pharmacy, Qassim University, Qassim, Saudi Arabia; ^7^Department of Laboratory and Blood Bank, King Fahad Specialist Hospital, Qassim, Saudi Arabia; ^8^Department of Microbiology, King Fahad Hospital, Al-Baha, Saudi Arabia; ^9^Department of Clinical Microbiology, King Abdulaziz University Hospital, Jeddah, Saudi Arabia; ^10^Department of Laboratory and Blood Bank, King Fahad Specialist Hospital, Tabuk, Saudi Arabia; ^11^Department of Microbiology, King Fahad Specialist Hospital, Tabuk, Saudi Arabia; ^12^Department of Microbiology, Maternity and Children Hospital, King Salman Medical City, Madinah, Saudi Arabia; ^13^Department of Natural Products, Faculty of Pharmacy, King Abdulaziz University, Jeddah, Saudi Arabia; ^14^Centre for Artificial Intelligence in Precision Medicines, King Abdulaziz University, Jeddah, Saudi Arabia

**Keywords:** *Pseudomonas aeruginosa*, antimicrobial resistance, broth microdilution, surveillance, Saudi Arabia, antibiotics

## Abstract

**Background:**

*Pseudomonas aeruginosa* is a common pathogen causing healthcare-associated infections. Most surveillance studies from Saudi Arabia that assessed the resistance by *P. aeruginosa* were conducted in single centers or did not use broth microdilution (BMD), the gold standard test. This is the first national multicenter study to assess the resistance profiles of *P. aeruginosa* isolates in Saudi Arabia using BMD.

**Methods:**

Between 2022 and 2023, isolates from various infection sites were collected from seven hospitals in seven different regions of Saudi Arabia. The isolates were shipped to an academic microbiology lab, where their susceptibility was tested by BMD following Clinical Laboratory Standards Institute guidelines using Sensititre GNX3F plates. %Susceptibility to each antibiotic, and MIC50 and MIC90 were determined.

**Results:**

In total, 185 *P. aeruginosa* isolates were collected. Most isolates came from respiratory specimens (34.1%), followed by urine (21.1%) and skin/soft tissue (17.8%). The highest susceptibility was to amikacin (76.8%). Concurrently, susceptibility to meropenem was 52%, but it was 43.8% to colistin. While all *P. aeruginosa* isolates met the definition of multidrug-resistance, 41 (22.2%) were difficult-to-treat and 10 (5.4%) were pandrug-resistant. Difficult-to-treat isolates made up 30.3% of skin and soft tissue isolates, 25.4% of respiratory isolates, 21.7% of blood isolates, and 17.9% of urine isolates.

**Conclusion:**

*Pseudomonas aeruginosa* demonstrated an unexpectedly high level of resistance to several commonly used antibiotics. Therefore, antimicrobial stewardship and infection control policies should be strictly enforced by hospitals across the country to optimize treatment, prevent the emergence and spread of resistant strains, and track resistance trends with local antibiograms.

## 1 Introduction

*Pseudomonas aeruginosa* is a common healthcare-associated bacterial pathogen associated with various severe infections, including pneumonia, bloodstream infections, and surgical site infections. *P. aeruginosa* is intrinsically resistant to several antimicrobial classes and can develop resistance to antibiotics typically used for treatment upon exposure (1). Various mechanisms allow *P. aeruginosa* to evade antibiotic activity and confer resistance, namely, enzyme production, blockade of antibiotic entry by narrowing porins, efflux of the antibiotic upon successful cellular entry, quorum sensing, and biofilm formation (1). The current methods used in hospitals to control biofilms, such as cleaning, disinfection, and surface preparation, demonstrated some effectiveness. However, these approaches still inadequate in achieving biofilm-related infections often re-emerge after treatment (2, 3).

Several terms were developed to describe *P. aeruginosa* isolates that are resistant to multiple antibiotics (4, 5). *P. aeruginosa* infections caused by such drug-resistant isolates are frequently linked to increased rates of morbidity and mortality as well as longer hospital stays, all of which add to the overall financial burden (1).

A recent review that included 11 surveillance studies of *P. aeruginosa* in Saudi Arabia over the last 10 years (2013–2023) revealed that most of the 8,995 *P. aeruginosa* isolates were susceptible to tobramycin (80%), followed by colistin (79.25%) and amikacin (63.85%) (6). Susceptibilities to antipseudomonal *β*-lactams and fluoroquinolones ranged between 33 and 60%. Notably, all the studies reported in this review used an automated susceptibility testing system rather than the broth microdilution (BMD) method, which is the gold standard of testing in surveillance studies (7–9). Larg-scale multicentric surveillance studies evaluating antimicrobial resistance (AMR) in Saudi Arabia are scarce. Additionally, the studies included in the 10-year review of *P. aeruginosa* susceptibilities in Saudi Arabia included isolates from a single center and/or used methods other than BMD (6).

Due to the lack of nationwide antimicrobial resistance studies in Saudi Arabia that includes more than one hospital and utilize BMD, we aimed to fill the gap by conducting the first largest multicenter surveillance study in Saudi Arabia to evaluate the susceptibility of a large number of *P. aeruginosa* isolates collected from seven different hospitals and to identify different drug resistance profiles. The study’s findings should inform decisions by policymakers and healthcare providers to tackle the issue of AMR based on the current susceptibilities of *P. aeruginosa* to various antibiotics and the prevalence of different drug-resistance patterns.

## 2 Materials and methods

### 2.1 Study period and collection process

Clinical isolates of *P. aeruginosa* from different infection sites were collected from seven participating large hospitals located in seven different administrative regions of Saudi Arabia from November 2022 to April 2023. The isolates were shipped to an academic microbiology laboratory (at the Faculty of Pharmacy, King Abdulaziz University, Jeddah) on appropriate agar plates. The study protocol was approved by the Research Ethics Committee of King Abdulaziz University (ref no. 559-22) and the Saudi Ministry of Health (reference no. 607/44/5113), both of which waived the need for informed consent.

### 2.2 Bacterial isolates

The participating hospitals collected consecutive clinical isolates of *P. aeruginosa* and labeled them with the date and the infection site. Identification of isolates was performed at each participating site using automated methods, such as VITEK 2 (bioMérieux, France) or BD Phoenix 100 (Becton, Dickinson and Company, NJ, USA). Once received, the isolates were immediately subcultured on blood agar plates (BAPs), which was followed by storage in cryovials containing glycerol at −80°C testing. The day before the testing days, the isolates were thawed, subcultured on BAPs, and incubated at 36°C for 24 h.

### 2.3 Antimicrobial susceptibility testing

BMD was conducted following Clinical Laboratory Standards Institute (CLSI) guidelines (M100-33) (8). Sensititre GNX3F plates (Thermo Fisher Scientific, USA) were employed for all the susceptibility tests, which include a panel of different antibiotics active against Gram-negative bacteria. The first microtiter plate of the batch was tested using the *P. aeruginosa* quality control isolate ATC27853 to ensure its accuracy. On the testing day, the microtiter plates were initially filled with 50 μL of prepared Mueller-Hinton Broth (Biolab, Inc., Hungary). Meanwhile, a bacterial inoculum of 0.5 McFarland standard (equivalent to 1.5 × 10^8^ CFU/mL) was prepared. The density was confirmed using a densitometer (Grant Instruments, Ltd., Shepreth, UK) that was calibrated on every testing day. The inoculum was instilled at a volume of 10 μL in the microtiter plates, which were then incubated at 36°C for 16–24 h prior to reading.

### 2.4 Definitions of drug resistance terms

Isolates that are resistant to three or more antimicrobial from different classes are labeled multidrug-resistant (MDR). In contrast, isolates that are specifically resistant to antipseudomonal *β*-lactams (piperacillin/tazobactam, ceftazidime, cefepime, aztreonam, meropenem, and imipenem/cilastatin) and fluoroquinolones (ciprofloxacin and levofloxacin) are labeled difficult-to-treat (DTR) (4, 5). Resistance to all antimicrobial from all classes renders an isolate pandrug-resistant (PDR) (5).

### 2.5 Data analysis

Minimum inhibitory concentration (MIC) results that were translated to susceptibility results based on CLSI breakpoints were input in Microsoft Excel (Microsoft, Corp., Seattle, WA, USA) to calculate the percentages of susceptible organisms (% susceptibility) to each tested antibiotic, MIC_50_, MIC_90_, and percentages of different drug resistance patterns.

## 3 Results

### 3.1 Characteristics and sources of the isolates

Of the seven participating hospitals, 185 *P. aeruginosa* isolates were collected. The characteristics of the hospitals and the distribution of the collected isolates are listed in Table 1, where the majority of the isolates were from the southern region (total *n* = 66), followed by the eastern region (*n* = 45), the western region (total *n* = 35), and the central region (*n* = 33).

**Table 1:** Characteristics of the included hospitals and the number of isolates collected.

Characteristic	Hospital						
	King Abdulaziz University Hospital	King Fahad Hospital of the University	King Khaled Hospital	King Fahad Specialist Hospital	King Fahad Hospital	King Fahad Specialist Hospital	Maternity and Children Hospital of King Salman Medical City
City	Jeddah	Dammam	Najran	Qassim	Albaha	Tabuk	Madinah
Region	Western	Eastern	Southern	Central	Southern	Northern	Western
Bed capacity	879	633	330	600	320	205	500
Number of ICUs	5 (99 beds)	5 (77 beds)	4 (56 beds)	4 (49 beds)	4 (64 beds)	2 (43 beds)	3 (117 beds)
N (%) of isolates	30 (16.2)	45 (24.3)	34 (18.4)	33 (17.8)	32 (17.3)	6 (3.2)	5 (2.7)

More than one-third of the isolates were obtained from respiratory specimens (*n* = 63; 34.1%). The second most common infection site was the urinary tract with 39 (21.1%) of the isolates, followed by skin/soft tissue and blood with 33 (17.8%) and 23 (12.4%) isolates, respectively (Table 2).

**Table 2:** Sources of *Pseudomonas aeruginosa* isolates (*n* = 185).

Source	*n* (%)
Respiratory	63 (34.1)
Urine	39 (21.1)
Skin or soft tissue	33 (17.8)
Blood	23 (12.4)
Ear	12 (6.5)
Body fluid	6 (3.2)
Vagina	4 (2.2)
Eye	3 (1.6)
Catheter tip	2 (1.1)

### 3.2 Antimicrobial susceptibility results

The susceptibility data of the isolates to the tested antibiotics, as well as MIC_50_, MIC_90_, and MIC ranges are listed in Table 3. The aminoglycosides class had the highest activity (%susceptibility ranging from 57.3 to 76.8%) compared with *β*-lactams, fluoroquinolones, and polymyxins. The highest susceptibility rate was exhibited to amikacin at 76.8%, whereas the lowest susceptibility was shown to ticarcillin/clavulanic acid at only 4.3%. Carbapenems were more effective than penicillins and cephalosporins, where the β-lactam to which the isolates had the highest susceptibility was meropenem at 52%, followed by doripenem at 49.2% and imipenem at 48.6%. While 43.8% of the isolates were susceptible to colistin, only 15.1% were susceptible to polymyxin B. The MIC_90_ of all the tested antibiotics was at the highest tested concentrations (Table 3).

**Table 3:** Antibiotic susceptibility data of *Pseudomonas aeruginosa.*

Antibiotic	% Susceptibility	MIC_50_ (μg/mL)	MIC_90_ (μg/mL)	MIC range
Amikacin	76.8	≤4	>32	≤4 - >32
Aztreonam	25.9	>16	>16	≤2 - >16
Cefepime	40	8	>16	≤2 - >16
Ceftazidime	41.6	8	>16	≤1 - >16
Ciprofloxacin	36.8	2	>2	≤0.06 - >2
Colistin^*^	43.8	4	>4	0.5 - >4
Doripenem	49.2	2	>4	≤0.5 - >4
Gentamicin	57.3	4	>8	≤1 - >8
Imipenem	48.6	4	>8	≤1 - >8
Levofloxacin	40.5	4	>8	≤1 - >8
Meropenem	52	4	>8	≤1 - >8
Piperacillin/tazobactam	47	32/4	>64/4	≤8/4 - >64/4
Polymyxin B^*^	15.1	4	>4	0.5 - >4
Ticarcillin/clavulanic acid	4.3	>128/2	>128/2	≤16/2 - >128/2
Tobramycin	57.8	4	>8	≤1 - >8

MIC, minimum inhibitory concentration.

* The percentages presented are %intermediate since per Clinical and Laboratory Standards Institute M100 document includes intermediate and resistant breakpoints only, which are ≤ 2 and ≥ 4 μg/mL, respectively.

### 3.3 Antimicrobial resistance patterns

**Figure 1:**
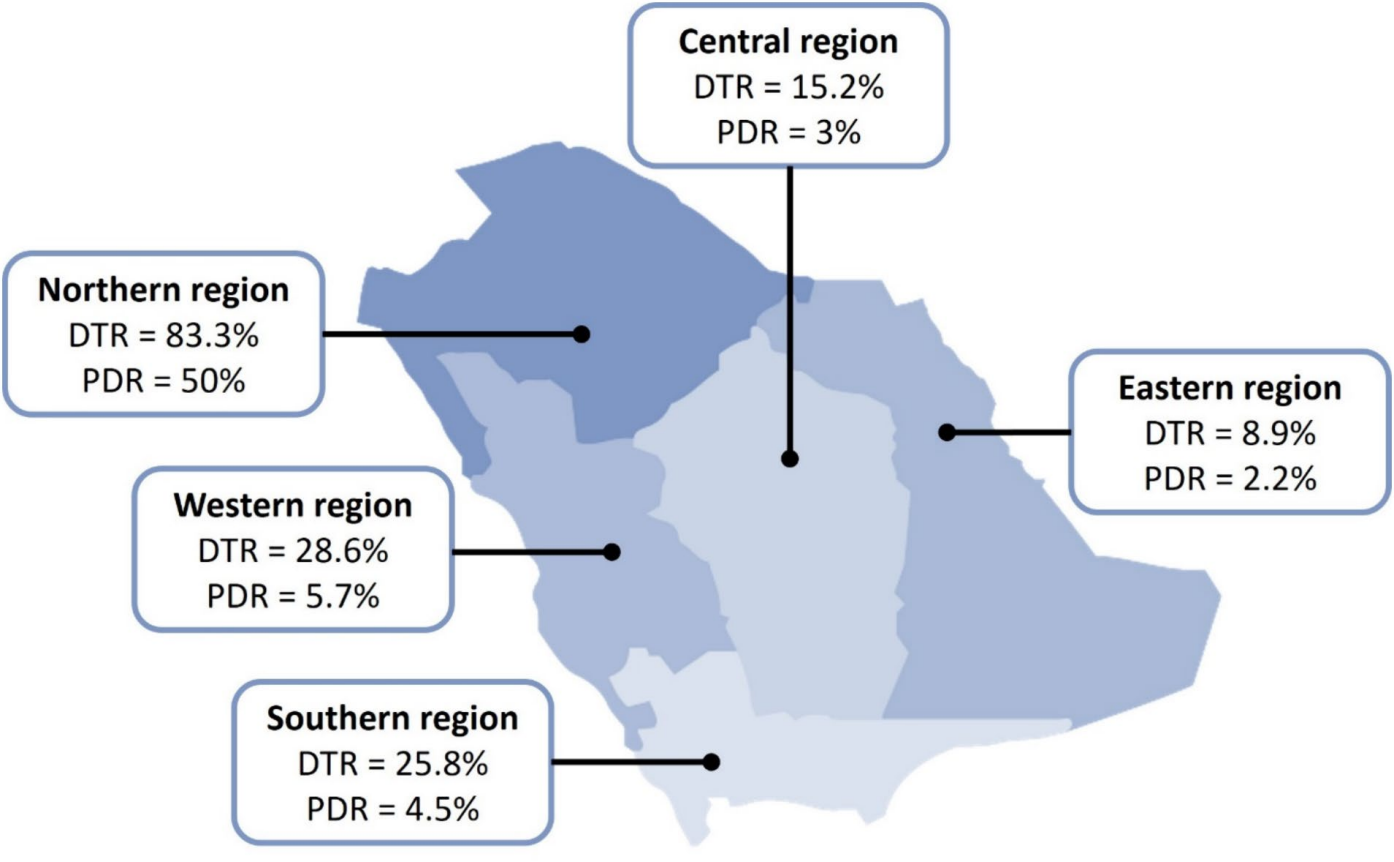
Distribution of DTR and PDR *Pseudomonas aeruginosa* in Saudi Arabia. DTR, difficult-to-treat; PDR, pandrug-resistant. All collected isolates were reported multidrug-resistant (MDR).

All *P. aeruginosa* isolates met the definition of MDR compared to 41 (22.2%) isolates that met the definition of DTR and 10 (5.4%) that were PDR. The distribution of the resistance patterns in different regions of Saudi Arabia is depicted in Figure 1. DTR isolates accounted for 16/63 (25.4%) of respiratory isolates, 10/33 (30.3%) of skin and soft tissue isolates, 7/39 (17.9%) of urine isolates, 5/23 (21.7%) of blood isolates, 2/6 (33.3%) of body fluid isolates, and 1/12 (8.3%) of ear isolates. In contrast, the most common sites of PDR isolates were ear (1/11; 8.3%), respiratory tract (4/63; 6.3%), skin and soft tissue (2/33; 6.1%), urine (2/39; 5.1%), and blood (1/23; 4.3%).

## 4 Discussion

This national surveillance study was performed in Saudi Arabia, where 185 unique *P. aeruginosa* isolates were tested for susceptibility to multiple antibiotics. The isolates were collected from seven different regions of the country and different infection sites. The most common infection sites from which the isolates were collected were the respiratory tract, urinary tract, skin and soft tissue, and blood. Notably, the susceptibility to aminoglycosides was higher than that to carbapenems and fluoroquinolones, and it was higher to carbapenems than to other *β*-lactams. Specifically, the highest susceptibility was recorded against amikacin with a susceptibility rate of 76.8%, whereas the lowest susceptibility rate was against ticarcillin/clavulanic acid at 4.3%. MIC ranges of all the tested antibiotics ranged from susceptible values to beyond the resistance breakpoints established by CLSI.

Most isolates in our study were isolated from respiratory (34.1%), urinary (21.1%), skin and soft tissue (17.8%), and blood (12.4%) specimens, indicating that the most common infections caused by *P. aeruginosa* were pneumonia, urinary tract infections (UTIs), skin and soft tissue infections (SSTIs), and bacteremia. A recent review by Reynolds et al. on the epidemiology of *P. aeruginosa* nosocomial infections corroborated these findings of the most common infections caused by *P. aeruginosa* while implying that the predominant reason for pseudomonal SSTIs is surgical site infections (10). Based on these findings, appropriate hospital infection control measures should be enforced to reduce the burden of nosocomial *P. aeruginosa* infections, particularly in operation rooms. Additionally, patients developing hospital-acquired pneumonia should be promptly treated empirically with regimens involving antipseudomonal agents to which *P. aeruginosa* exhibit high susceptibility rates according to the hospital’s antibiogram (11).

Several surveillance studies from Saudi Arabia reported different resistance rates by *P. aeruginosa* to different antibiotics over the past 10 years (6). The high susceptibility rate to amikacin shown in our study (76.8%) was consistent with observations from previous studies, where susceptibility rates to amikacin reached 81% to more than 90% in major hospitals from the Hail region (in the northern part), the southern region, Riyadh (central region), and the western region (12–16). A much lower susceptibility rate of 50% was observed in a hospital from Hafr Al-Batin (northern region; not included in the current study) (17). Although the susceptibility to gentamicin was reported in the study, recent recommendations from CLSI in 2023 eliminated gentamicin as a treatment option for *P. aeruginosa* (18). This is due to the inability of gentamicin to achieve bacteriostasis (rather than bactericidal activity) only if gentamicin MIC was ≤0.5 μg/mL, which is far below the previous MIC susceptibility breakpoint of ≤4 μg/mL reported in the M100-32 document of CLSI in 2022, and far below the epidemiological cutoff value of 8 μg/mL (18). Such observations raised the concern of potential intrinsic resistance and that increasing gentamicin dose to overcome this issue may put patients at high risk of nephrotoxicity. Furthermore, the recent recommendations by CLSI called to confine the use of amikacin against *P. aeruginosa* to UTIs; thus, microbiology laboratories are advised to report amikacin MIC only for *P. aeruginosa* isolated from the urine (18).

Antipseudomonal *β*-lactams are widely used empirically to treat infections presumed to be caused by *P. aeruginosa*. In this study, carbapenems had the highest activity (48.6–52%) followed by piperacillin/tazobactam (47%) and cephalosporins (40–41.8%). Among the carbapenems, the susceptibility was the highest to meropenem (52%), followed by doripenem (49.2%) and imipenem (48.2%). Meropenem and imipenem are commonly used in Saudi Arabia unlike doripenem, which is not commercially available as it is not registered by the Saudi Food and Drug Authority. The studies from Hail and Riyadh demonstrated comparable susceptibility rates to meropenem of 56 and 58.9%, respectively (12, 19). The susceptibility rates to imipenem were in line with those studies, with a rate of around 50% in Hafr Al-Batin Hail (12, 17). Nevertheless, much lower susceptibility rates of 19–22.2% and were reported in a study from Riyadh (central region) and the western region (14, 16). Our study did not assess the susceptibility to ceftolozane/tazobactam, ceftazidime/avibactam, and cefiderocol, which are novel cephalosporins, because they were not included in the prefilled microtiter plates used. Nonetheless, a recent global surveillance study by the ERACE-PA Global Study Group assessed the susceptibility of *P. aeruginosa* isolates, which included 28 isolates from one of the hospitals enrolled in our study (King Abdulaziz University Hospital in Jeddah), between 2019 and 2021 to ceftolozane/tazobactam and ceftazidime/avibactam (20). This investigation revealed that isolates from the Middle East exhibited susceptibility rates of 47 and 57% to these agents, respectively. Conversely, the susceptibility rate to cefiderocol was 97% as reported in another study that evaluated the same collection of isolates (21).

The susceptibility rates to other commonly used antipseudomonal antibiotics were below optimum. For instance, the susceptibility to piperacillin/tazobactam, ceftazidime, cefepime, levofloxacin, and ciprofloxacin were 47, 41.6, 40, 40.5, and 36.8%, respectively. In the recent 10-year review, the collective average susceptibility rates to these antibiotics were comparable ranging from 40.2% to levofloxacin to as high as 60.3% to piperacillin/tazobactam (6). A comparable susceptibility rate to cefepime (42%) was reported for the isolates collected from the Middle East from the ERACE-PA global surveillance study. However, a lower susceptibility was observed to ceftazidime compared to our findings (33% vs. 41.6%) (20). The latter observation could be attributed to the involvement of hospitals from the Middle East region other than Saudi Arabia as the total number of isolates collected from the Middle East was 163, of which only 28 were from Saudi Arabia.

In our study, some *P. aeruginosa* isolates were resistant to carbapenems while being susceptible to other antipseudomonal *β*-lactams. This observation indicates that carbapenem resistance by *P. aeruginosa* is not limited to the production of inhibitory enzymes but it also involves different mechanisms, such as decreased porins permeability and antibiotic efflux (1). Gill et al. developed an algorithm to guide carbapenemase testing in carbapenem-resistant *P. aeruginosa* (22). In their study, the isolates that were resistant to all antipseudomonal β-lactams, despite their susceptibility to ceftolozane/tazobactam and ceftazidime/avibactam, were genotypically tested for carbapenemase production. The authors discovered that the presence of carbapenem resistance along with non-susceptibility to cefepime, ceftazidime, and piperacillin/tazobactam is an indicator for carbapenemase production testing genotypically. Moreover, the algorithm was validated on the isolates collected by the ERACE-PA Global Study Group, which resulted in a 43% reduction in the need for carbapenemase production testing compared to testing all carbapenem-resistant isolates that exhibit susceptibility to other antipseudomonal *β*-lactams (23). Among the isolates of our cohort, 41 of 185 (22.2%) met the algorithm’s criteria for carbapenemase testing, implying that only one-fifth of the isolates could be carbapenemase-producing. In the Middle East, the most commonly reported carbapenemases produced by *P. aeruginosa* were Verona imipenemase (VIM), Guiana-extended-spectrum (GES), and imipenemase (IMP), as noted in the global surveillance study at rates of 37, 24, and 17%, respectively (20).

Colistin is often used as a last-line option in the absence of safer effective alternatives given its poor safety profile of nephrotoxicity and neurotoxicity. In the current study, we found a relatively low susceptibility rate to colistin at 43.8%. However, other studies conducted in different regions of Saudi Arabia reported varying susceptibility rates for colistin ranging 44.4–91% (15, 24). Despite its nephrotoxicity, colistin remains a viable option against tough Gram-negative bacteria; however, the increasing resistance against it is highly concerning (24). The average collective susceptibility rate to colistin from studies published within the past 10 years was 79.25%. Nonetheless, all the prior studies that evaluated the susceptibility to colistin utilized automated systems instead of BMD, which is recommended by the latest international colistin guidelines (25). Notably, polymyxin B exhibited extremely poor activity with a susceptibility rate of 15.1% despite not being used in Saudi Arabia as it is not registered by the Saudi Food and Drug Authority. The low susceptibility rates to colistin and polymyxin B could be explained by the findings of two previous BMD studies that noticed that polymyxins, including colistin and polymyxin B, exhibit affinity to plastic, the material, which is the material used to make BMD microtiter plates (26, 27). The presence of such an effect was found to impact MIC measurements. Therefore, Sutherland et al. recommended adding polysorbate 80 to the testing wells of the microtiter plates when testing colistin MIC using BMD as polysorbate 80 acts as a surfactant that would prevent the adherence of colistin to the plastic bottom of the plate. In our study, the low susceptibility rate to colistin could be potentially attributed to this phenomenon. Since the plates used in our study were prefilled with lyophilized antibiotics, the addition of polysorbate 80 was not possible.

The term DTR *P. aeruginosa* has been recently reported in the latest guidelines on the management of infections caused by antimicrobial resistant Gram-negative bacteria (4). In our study, more than one-fifth of the isolates (22.2%) met the DTR definition (i.e., resistant to all antipseudomonal *β*-lactams and fluoroquinolones) and only 5.4% were PDR. Although these low rates may sound encouraging, it should be noted that all the isolates were considered MDR, which could be alarming and should encourage policymakers in hospitals to enforce antimicrobial stewardship and infection control. Furthermore, clinicians should be educated about the appropriate use of antibiotics and the judicious use of broad-spectrum antibiotics, particularly carbapenems and colistin, when less broad-spectrum agents, such as ceftazidime, can be used. These efforts collectively should help control the spread of resistant *P. aeruginosa* and prevent a surge in DTR and PDR rates. Of note, the highest rates of DTR and PDR were reported from the northern region. This inflation could be due to a numerical effect as only six isolates were sent from that region compared with the other four regions.

In this study, we conducted a nationwide investigation from different regions of Saudi Arabia resulting in a larger number of isolates. These specific regions could have distinct patterns of antimicrobial usage, leading to variations in susceptibility rates. Although our intention was to incorporate additional hospitals into the study to expand the sample size, some logistical issues with certain hospitals hindered our efforts. Additionally, the prefilled microtiter plates lacked ceftolozane/tazobactam, ceftazidime/avibactam, and cefiderocol, which are essential antibiotics used clinically against MDR *P. aeruginosa*. Lastly, performing genotypic testing in a future study is recommended to gain a better understanding of the resistance mechanisms at play especially the hazardous DTR and PDR isolates.

## 5 Conclusion

Antibiotic treatment plans for *P. aeruginosa* infections in Saudi Arabia can be customized considering data derived from this study, which showed a high rate of MDR isolates and alarming rates of DTR and PDR isolates, including average susceptibilities to broad-spectrum antibiotics, such as carbapenems and colistin. However, though the use of local institutional antibiogram is preferred, which can lower the chance of treatment failure and improve patient outcomes. While we included a relatively high number of isolates from various regions of Saudi Arabia, the results may not necessarily be generalizable to all the hospitals in the country. The information derived from our study is crucial for policymakers to make informed decisions regarding the development of strategies to combat AMR. Additionally, healthcare providers can tailor their treatment approaches based on the prevailing susceptibility patterns. The data generated from this research will also serve as a valuable resource for guiding public health initiatives, formulating antimicrobial stewardship programs, and ultimately improving patient outcomes in the face of emerging antibiotic resistance challenges.

## Data Availability

The raw data supporting the conclusions of this article will be made available by the authors, without undue reservation.

## References

[1] Thabit AK Crandon JL Nicolau DP. Antimicrobial resistance: impact on clinical and economic outcomes and the need for new antimicrobials. Expert Opin Pharmacother. (2015) 16:159–77. doi: 10.1517/14656566.2015.993381, PMID: 25496207

[2] Elfaky MA. Unveiling the hidden language of bacteria: anti-quorum sensing strategies for gram-negative bacteria infection control. Arch Microbiol. (2024) 206:124. doi: 10.1007/s00203-024-03900-0, PMID: 38409503

[3] Preda VG Sandulescu O. Communication is the key: biofilms, quorum sensing, formation and prevention. Discoveries. (2019) 7:e100:e10. doi: 10.15190/d.2019.13, PMID: 32309618 PMC7086079

[4] Tamma PD Aitken SL Bonomo RA Mathers AJ van Duin D Clancy CJ. Guidance on the treatment of antimicrobial resistant gram-negative infections. Clin Infect Dis. (2023) 2023:ciad428. doi: 10.1093/cid/ciad428, PMID: 37463564

[5] Magiorakos AP Srinivasan A Carey RB Carmeli Y Falagas ME Giske CG. Multidrug-resistant, extensively drug-resistant and pandrug-resistant bacteria: an international expert proposal for interim standard definitions for acquired resistance. Clin Microbiol Infect. (2012) 18:268–81. doi: 10.1111/j.1469-0691.2011.03570.x, PMID: 21793988

[6] Thabit AK Alabbasi AY Alnezary FS Almasoudi IA. An overview of antimicrobial resistance in Saudi Arabia (2013-2023) and the need for National Surveillance. Microorganisms. (2023) 11:2086. doi: 10.3390/microorganisms11082086, PMID: 37630646 PMC10460018

[7] Engelkirk Paul G. Duben-Engelkirk Janet L.. Laboratory diagnosis of infectious diseases: essentials of diagnostic microbiology. Baltimore, MD: Lippincott Williams & Wilkins. p. 168–169. (2008).

[8] Clinical and Laboratory Standards Institute. Performance standards for antimicrobial susceptibility testing: thertieth informational supplement [document M100-S33]. Wayne, PA: National Committee for Clinical Laboratory Standards (2023).

[9] European Committee for Antimicrobial Susceptibility Testing (EUCAST) of the European Society of Clinical Microbiology and Infectious Diseases (ESCMID). Determination of minimum inhibitory concentrations (MICs) of antibacterial agents by broth dilution. Clin Microbiol Infect. (2003) 9:ix–xv. doi: 10.1046/j.1469-0691.2003.00790.x 11168187

[10] Reynolds D Kollef M. The epidemiology and pathogenesis and treatment of *Pseudomonas aeruginosa* infections: an update. Drugs. (2021) 81:2117–31. doi: 10.1007/s40265-021-01635-6, PMID: 34743315 PMC8572145

[11] Kalil AC Metersky ML Klompas M Muscedere J Sweeney DA Palmer LB. Executive summary: management of adults with hospital-acquired and ventilator-associated pneumonia: 2016 clinical practice guidelines by the infectious diseases society of America and the American thoracic society. Clin Infect Dis. (2016) 63:575–82. doi: 10.1093/cid/ciw504, PMID: 27521441 PMC4981763

[12] Said KB Alsolami A Khalifa AM Khalil NA Moursi S Osman A. A multi-point surveillance for antimicrobial resistance profiles among clinical isolates of gram-negative bacteria recovered from major Ha'il hospitals, Saudi Arabia. Microorganisms. (2021) 9:2024. doi: 10.3390/microorganisms9102024, PMID: 34683344 PMC8537776

[13] Ibrahim ME. High antimicrobial resistant rates among gram-negative pathogens in intensive care units. A retrospective study at a tertiary care hospital in Southwest Saudi Arabia. Saudi Med J. (2018) 39:1035–43. doi: 10.15537/smj.2018.10.22944, PMID: 30284588 PMC6201019

[14] Alshammari HO Somily A Yahia Qattan M Alsubki RA Moussa IM. Susceptibility pattern of multi-drug resistance *Pseudomonas aeruginosa* isolates from tertiary care hospital in Riyadh, KSA. J King Saud Univ Sci. (2023) 35:102702. doi: 10.1016/j.jksus.2023.102702

[15] Hafiz TA Bin Essa EA Alharbi SR Alyami AS Alkudmani ZS Mubaraki MA. Epidemiological, microbiological, and clinical characteristics of multi-resistant *Pseudomonas aeruginosa* isolates in King Fahad Medical City, Riyadh, Saudi Arabia. Trop Med Infect Dis. (2023) 8:205. doi: 10.3390/tropicalmed8040205, PMID: 37104331 PMC10145365

[16] Khan MA Faiz A. Antimicrobial resistance patterns of *Pseudomonas aeruginosa* in tertiary care hospitals of Makkah and Jeddah. Ann Saudi Med. (2016) 36:23–8. doi: 10.5144/0256-4947.2016.23 26922684 PMC6074268

[17] Al Yousef SA. Surveillance of antibiotic-resistant bacteria in King Khalid Hospital, Hafr Al-Batin, Saudi Arabia, during 2013. Jundishapur J Microbiol. (2016) 9:e19552. doi: 10.5812/jjm.19552, PMID: 27942366 PMC5136423

[18] Clinical and Laboratory Standards Institute. AST news update June 2023: new! CLSI M100-Ed33: updated aminoglycoside breakpoints for *Enterobacterales* and *Pseudomonas aeruginosa*. Malvern, PA: Clinical and Laboratory Standards Institute (2023).

[19] Alhussain FA Yenugadhati N Al Eidan FA Al Johani S Badri M. Risk factors, antimicrobial susceptibility pattern and patient outcomes of *Pseudomonas aeruginosa* infection: a matched case-control study. J Infect Public Health. (2021) 14:152–7. doi: 10.1016/j.jiph.2020.11.010, PMID: 33422856

[20] Gill CM Aktaþ E Alfouzan W Bourassa L Brink A Burnham C-AD. The ERACE-PA global surveillance program: ceftolozane/tazobactam and ceftazidime/avibactam in vitro activity against a global collection of Carbapenem-resistant *Pseudomonas aeruginosa*. Eur J Clin Microbiol Infect Dis. (2021) 40:2533–41. doi: 10.1007/s10096-021-04308-0, PMID: 34291323 PMC8590662

[21] Gill CM Santini D Nicolau DP Group E-PGS. *In vitro* activity of cefiderocol against a global collection of carbapenem-resistant *Pseudomonas aeruginosa* with a high level of carbapenemase diversity. J Antimicrob Chemother. (2024) 79:412–6. doi: 10.1093/jac/dkad396, PMID: 38153232 PMC10832583

[22] Gill CM Asempa TE Nicolau DP. Development and application of a pragmatic algorithm to guide definitive carbapenemase testing to identify carbapenemase-producing *Pseudomonas aeruginosa*. Antibiotics. (2020) 9:738. doi: 10.3390/antibiotics9110738, PMID: 33120865 PMC7693613

[23] Gill CM Aktathorn E Alfouzan W Bourassa L Brink A Burnham CD. Multicenter, prospective validation of a phenotypic algorithm to guide carbapenemase testing in carbapenem-resistant *Pseudomonas aeruginosa* using the ERACE-PA global surveillance program. Open Forum Infect Dis. (2022) 9:ofab617. doi: 10.1093/ofid/ofab617 35106312 PMC8801223

[24] Eljaaly K Bidell MR Gandhi RG Alshehri S Enani MA Al-Jedai A. Colistin nephrotoxicity: meta-analysis of randomized controlled trials. Open Forum Infect Dis. (2021) 8:ofab026. doi: 10.1093/ofid/ofab026, PMID: 33623807 PMC7888569

[25] Tsuji BT Pogue JM Zavascki AP Paul M Daikos GL Forrest A. International consensus guidelines for the optimal use of the polymyxins: endorsed by the American College of Clinical Pharmacy (ACCP), European Society of Clinical Microbiology and Infectious Diseases (ESCMID), Infectious Diseases Society of America (IDSA), International Society for Anti-infective Pharmacology (ISAP), Society of Critical Care Medicine (SCCM), and Society of Infectious Diseases Pharmacists (SIDP). Pharmacotherapy. (2019) 39:10–39. doi: 10.1002/phar.2209, PMID: 30710469 PMC7437259

[26] Sharafi T Ardebili A. Plastic binding feature of polymyxins: the effect on MIC susceptibility measurements. Infect Drug Resist. (2019) 12:2649–53. doi: 10.2147/IDR.S219130, PMID: 31695440 PMC6717857

[27] Sutherland CA Nicolau DP. To add or not to add polysorbate 80: impact on colistin MICs for clinical strains of *Enterobacteriaceae* and *Pseudomonas aeruginosa* and quality controls. J Clin Microbiol. (2014) 52:3810. doi: 10.1128/JCM.01454-14, PMID: 25210064 PMC4187774

